# Antioxidant effects of compound walnut oil capsule in mice aging model induced by D-galactose

**DOI:** 10.29219/fnr.v62.1371

**Published:** 2018-04-20

**Authors:** Huandong Zhao, Jian Li, Juan Zhao, Yang Chen, Caiping Ren, Yuxiang Chen

**Affiliations:** 1 Key Laboratory of Nanobiological Technology of Chinese Ministry of Health, Xiangya Hospital, Central South University, Changsha, China; 2 School of Pharmaceutical Sciences, Central South University, Changsha, China; 3 Institute of Biomedical Engineering, Xiangya Hospital, Central South University, Changsha, China; 4 Department of Clinical Laboratory, Xiangya Hospital, Central South University, Changsha, China; 5 Cancer Research Institute, Collaborative Innovation Center for Cancer Medicine, Key Laboratory for Carcinogenesis of Chinese Ministry of Health, School of Basic Medical Science, Central South University, Changsha, China

**Keywords:** walnut oil capsule, oxidative stress, antioxidant, D-galactose, aging model

## Abstract

**Background:**

Many plant original foods have been shown beneficial effects in humans. In the previous work, we have developed a compound capsule which contains major constituents of walnut oil and grape seed extract.

**Objective:**

To investigate the antioxidant effects of the Compound Walnut Oil Capsule (WOC) in aging model induced by D-gal.

**Design:**

70 C57BL/6J mice were randomly divided into seven groups. Mice in normal group received daily subcutaneous injection of saline while the control group, WOC groups, Vitamin C (VC) group and pure walnut oil group received daily subcutaneous injection of D-galactose (D-gal) for 8 weeks. Total antioxidant capacity (T-AOC), super dismutase (SOD), glutathione peroxidase (GSH-Px) and malondialdehyde (MDA) in serum, liver and brain were determined. The expression of Heme Oxygenase (HO-1), iNOS and Klotho in liver and brain were obtained.

**Results:**

WOC could improve the pathologic lesions caused by oxidative stress and significantly enhance the T-AOC, increase the activities of SOD, GSH-Px and decrease the contents of MDA in serum, liver and brain. Also, the WOC could obviously up-regulate the expression of HO-1 and Klotho and down-regulate the expression of iNOS.

**Conclusion:**

WOC can be used as an anti-aging food for its effectively eliminating free radicals, enhancing the antioxidant capacity and alleviating the damages of oxidative stress.

**Figure:**
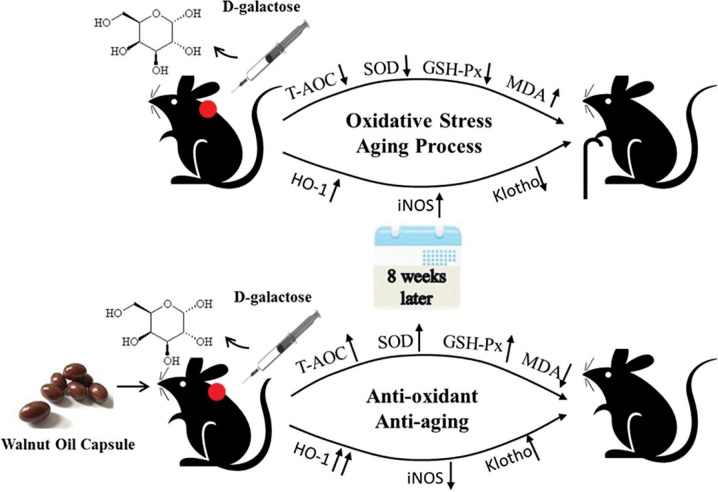

Aging is a complex natural process closely related to oxidative stress and free radicals, which has become a hot topic nowadays (1, 2). Accumulation of free radicals can affect the functions and abilities of human body such as lung, heart, and brain (3, 4). It is important to select appropriate scavengers to protect body from the damage of free radicals and improve the quality of life.

Natural drugs for their low toxicity and low side-effects are widely used in the field of medicine. It is reported that many plant original foods and medicines have potential antioxidant capacity *in vitro* and *in vivo*, such as *Asparagus cochinchinensis (Lour.),* (5) *Trollius chinensis Bunge* (6), and *Cinnamomum verum* (7). In the research, we have developed a compound capsule which contains major constituents of walnut oil and grape seed extract. Walnut oil is rich in linolenic acid and linoleic acid; previous studies showed that it has several health benefits including anti-inflammatory and antioxidant effects (8, 9). Many researches indicated that the main ingredients of grape seed extract are anthocyanin and procyanidin, which have strong anti-inflammatory and anti-lipid peroxidation effects (10–12).

Nowadays, the aging model induced by D-galactose (D-gal) is widely used in the field of oxidative stress and antioxidant research for its easy operation, cheapness, and short periods, which was first reported by Gong et al. in 1997 (13). Long-time injection of high dosage of D-gal can cause massive production of free radicals, neurotoxicity, tissue injury, and inflammation, followed by senescence (14, 15). The inbred C57BL/6J mice were widely used in the brain aging research for its lower growth rate and smaller individual difference (16). In our work, we used walnut oil capsule (WOC) to investigate its antioxidant effects in C57BL/6J mice aging model established by D-gal. Not only did we detect the activities of oxidative stress–related enzymes but also discussed the expression of some free radicals and aging-related enzymes at the molecular level. The histopathologic examination was also observed to see the effects of WOC on liver and brain. The research aimed to develop the WOC as an antioxidant health food for elderly people.

## Materials and methods

### Drugs and reagents

WOC was prepared by us according to designed prescription and the China patent (No:201410564056.x). Pure walnut oil was purchased from Valder Fields CO., Ltd (Yuxi, China), and Vitamin C (VC) from Solarbio Science & Technology CO., Ltd (Beijing, China). D-gal was purchased from Sigma-Aldrich (St Louis, MO, USA). 1,1-diphenyl-2-picrylhydrazyl (DPPH) was purchased from TCI (Shanghai) Development Co., Ltd (Shanghai, China). Total antioxidant capacity (T-AOC), superoxide dismutase (SOD), glutathione peroxidase (GSH-PX) and malondialdehyde (MDA) kits were purchased from Jiancheng Bioengineering Institute (Nanjing, China). BCA protein assay kit and SDS-PAGE kits were purchased from Multi Sciences Biotech CO., Ltd (Hangzhou, China). Anti-Heme Oxygenase-1 (HO-1) antibody and anti-Klotho antibody were purchased from Abcam (Shanghai, China). Anti-iNOS antibody and anti-β-actin antibody were purchased from cell signaling technology (Danvers, USA).

### Measurement of radical scavenging in vitro

DPPH free radicals scavenging test has been effectively used to evaluate the free radical scavenging capacity of antioxidant drugs *in vitro* (17). Briefly, 20 μL of sample was quickly mixed with 180 μL 0.1 mmol/L DPPH and placed in the dark for 0 min, 10 min, 20 min, and 30 min. Then the absorbance at 517 nm was measured. Absolute alcohol was used as negative control. The DPPH scavenging rate was determined using K% = [1-(Ai–Aj)/Ao] × 100% (Ai, the absorbance of sample mixed with DPPH; Aj, the absorbance of sample mixed with absolute alcohol; Ao, the absorbance of negative control).

### Treatment of animals

Seventy 3-month-old C57BL/6J male mice, weighing about 20 ± 2 g, were obtained from CAVENS Laboratory Animal Co., Ltd (Changzhou, China; certification No. SCXK (Su) 2016-0010). The mice were housed in an SPF environment at 25°C and 60% relative humidity under 12-/12-h dark/light cycle with free access to food and water. The protocols were conducted in accordance with the Guidance Suggestions for the Care and Use of Laboratory Animals, formulated by the Ministry of Science and Technology of China. After a week of acclimation, the mice were randomly divided into seven groups (10 mice/group), including a normal group; an aging model group as control; WOC low, medium, and high groups; a VC group; and a pure walnut oil group as positive control. For the control group, WOC groups, positive control groups, all mice received daily subcutaneous injection of 1,000 mg/kg D-gal for 8 weeks. For mice in normal group, 0.2 mL physiological saline was injected for 8 weeks. From the second week, mice in the WOC, positive control groups received daily intragastric administration of WOC at doses of 6 mL/kg, 12 mL/kg, 18 mL/kg; VC at a dose of 200 mg/kg; pure walnut oil at a dose of 12 mL/kg, while the normal and control groups received intragastric administration of distilled water at a dose of 12 mL/kg.Twenty-four hours after final administration, the mice were anaesthetized by intraperitoneal injection of 10 mL/kg 5% chloral hydrate; 0.5 mL blood samples were extracted from peri-orbital sinus and the serum was collected after being centrifuged at 3,000 r/min for 10 min. Mice were sacrificed, followed by rapid isolation of liver and brain.

### Histomorphological observation of liver and brain

Tissues were fixed in 10% formalin at room temperature for 48 h, then desiccated and embedded in paraffin. After that, tissues were sliced into 5 μm slices for hematoxylin-eosin (H&E) staining. Specimens were scanned and pathological changes were observed using Case Viewer 2.0 (3D HISTECH, Ltd, Budapest, Hungary).

### Western blotting

Western blot analysis was performed as described previously (18). Briefly, tissues were lysed into homogenate using RIPA lysis buffer and PMSF, and total proteins were separated by SDS-PAGE and then transferred to PVDF membranes at 300 mA for 1 h. After being transferred, the membranes were placed in blocking buffer (5% non-fat milk in Tris-buffered saline containing Tween-20 [TBST]) for 1 h, and the blots were incubated with an appropriate primary antibody (anti-Klotho antibody [1:1,000], anti-HO-1 antibody [1:20,000], anti-iNOS antibody [1:1,000], anti-β-actin antibody [1:1,000]) at 4°C overnight, and then treated with secondary antibody (1:5,000) at room temperature for 1 h. Then the chemiluminescent indicator was applied to membranes and specific proteins were detected by fluorchem device.

### Statistical analysis

All data were expressed as mean ± SD and analyzed using SPSS 18.0 software (SPSS Inc., Chicago, IL, USA). The significance of difference among groups was tested by one-way analysis of variance (ANOVA) test, and intergroup differences were compared using least-significant difference *t*-test.

## Results

### In vitro radical scavenging of WOC

**Fig. 1:**
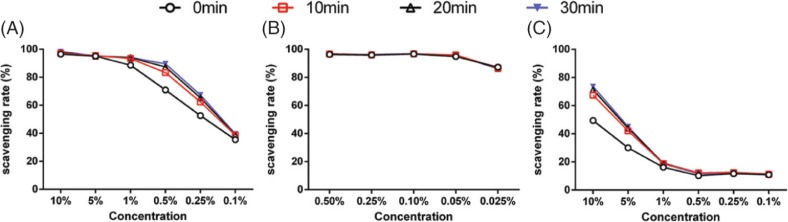
DPPH scavenging rates of WOC, VC, and pure walnut oil at different time points. (A) WOC; (B) VC; (C) Pure walnut oil.

The scavenging rate of WOC and pure walnut showed concentration dependence and time dependence. The WOC results showed that when the concentration was higher than 1%, the changes of scavenging rate was not obvious with the decrease of concentration, while when the concentration was lower than 1%, the scavenging rate decreased obviously. On the contrary, when the concentration was above 1%, pure walnut oil showed that the scavenging rate decreased sharply with the decrease of concentration, but it flattened out below 1%. As regards the result of VC, there was no obvious concentration dependence and time independence. Instead, the scavenging rate remained almost unchanged with the decrease in concentration, and as time went on, the scavenging rate was almost constant, which indicated the scavenging rate of VC reached the maximum in a short time after reaction. In other words, the DPPH scavenging rate followed VC > WOC > pure walnut oil *in vitro* (Fig. 1).

### General condition of mice

Mice were weighed daily and the weight changes were recorded in order to observe the effects of different drugs on mice body weight. Result showed that there were no significant differences among groups in body weight both on the first day and the last day (Table 1). But the growth rate in the normal group was higher than that in other groups (*p* < 0.05 or *p* < 0.01). It indicated that not only D-gal but also WOC, VC, and pure walnut oil could affect body weight in mice.

**Table 1:** The weight changes in mice

Group	First day (g)	Last day (g)	Growth rate (%)
Normal	25.21 ± 1.28	30.07 ± 1.35	19.80 ± 2.94
Control	25.53 ± 1.07	28.74 ± 1.81	12.51 ± 3.10**
WOC low	25.72 ± 1.19	29.49 ± 1.67	14.68 ± 4.18*
WOC medium	24.88 ± 1.61	28.43 ± 1.52	14.43 ± 4.68*
WOC high	25.19 ± 1.04	28.39 ± 1.70	12.44 ± 3.47**
VC	25.03 ± 1.76	28.57 ± 2.79	14.13 ± 7.81*
Pure walnut oil	25.12 ± 1.21	28.35 ± 1.90	12.78 ± 2.13*

The body weight change in C57BL/6J mice on the first and last day.

* *p* < 0.05

** *p* < 0.01 versus normal group (*n* = 10 in each group).

WOC, walnut oil capsule; VC, Vitamin C.

**Fig. 2:**
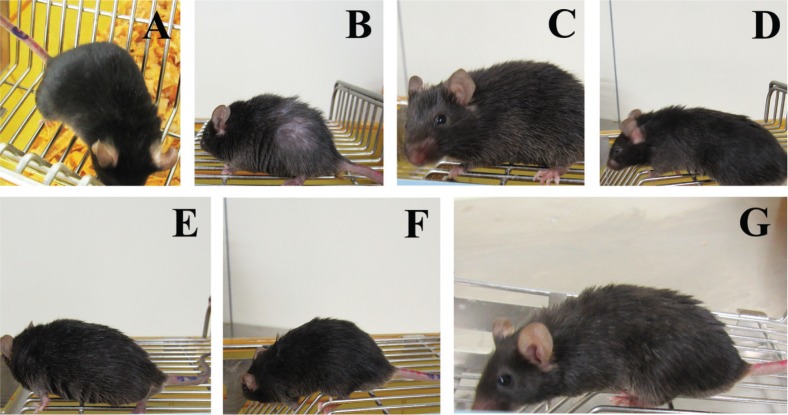
The appearance changes in different groups. (A) Normal group; (B) Control group; (C) WOC low group; (D) WOC medium group; (E) WOC high group; (F) VC group; (G) Pure walnut oil.

There were also differences in appearance among different groups (Fig. 2). Mice in the normal group had smooth hair and active spirit (A), but in the control group, mice were curled up and had coarse hair, severe hair loss, and poor spirit (B). Mice treated with WOC, VC, and pure walnut oil exhibited improvements in their hair color and spirit (C D E F G).

### The effects of WOC on histological changes in liver and brain

**Fig. 3:**
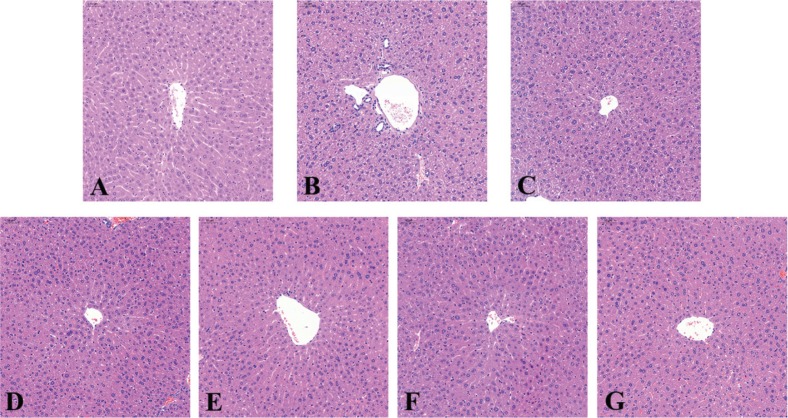
Pathological changes in liver. (A) Normal group; (B) Control group; (C) WOC low group; (D) WOC medium group; (E) WOC high group; (F) VC group; (G) Pure walnut oil.

The pathological changes in liver were shown in Fig. 3. In the normal group, the shape of cell nucleus was large and round. Hepatic cords were neatly arranged and there were no cell necrosis and degeneration. There were spotty necrosis, hydropic degeneration, vacuolar degeneration and lymphocytic infiltration in the control group. And the hepatic cords were disarranged. Mice treated with WOC, VC, and pure walnut oil had exhibited improvements in these situations. And as the dose of WOC increased, the improvements got better.

**Fig. 4:**
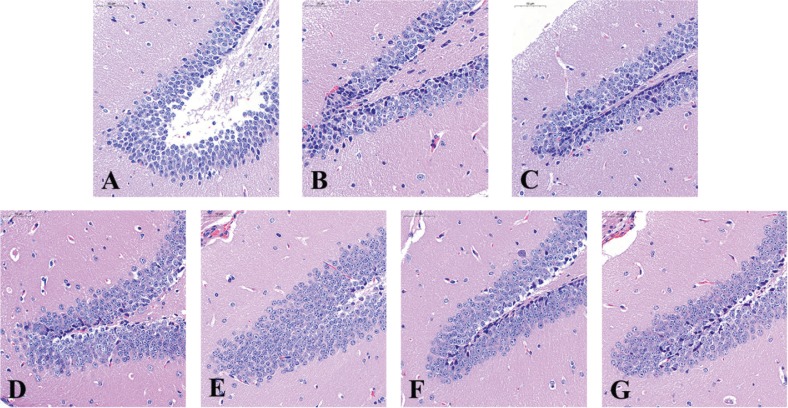
Pathological changes in brain (H&E staining, ×500). (A) Normal group; (B) Control group; (C) WOC low group; (D) WOC medium group; (E) WOC high group; (F) VC group; (G) Pure walnut oil.

The pathological changes in hippocampus dentate gyrus were observed (Fig. 4). Compared with the normal group, the granular cells in the control group were smaller, fewer, and disarranged. Furthermore, cells with hyperchromatism, changes of nuclear shape, and karyopyknosis were also observed, which indicated cell aging (19, 20). Groups treated with WOC, VC, and pure walnut oil showed improvements in the cells number and arrangement.

### The effects of WOC on T-AOC, SOD, GSH-Px, and MDA

**Fig. 5:**
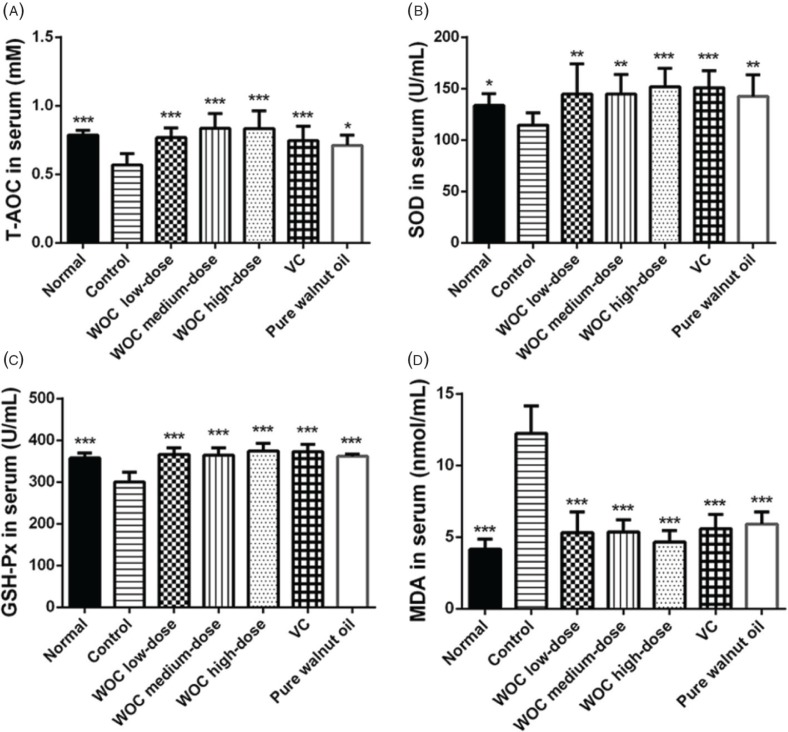
Biochemical indicators in serum. (A) Effects of WOC on T-AOC; (B) Effects of WOC on the activities of SOD; (C) Effects of WOC on the activities of GSH-Px; (D) Effects of WOC on the contents of MDA. *p < 0.05, **p < 0.01,*p < 0.001 versus the control group.

In the result of serum (Fig. 5), decreased levels of T-AOC, SOD, and GSH-Px, and increased contents of MDA were found in the control group compared with the normal group (*p* < 0.05 or *p* < 0.001). Mice treated with WOC, VC, and pure walnut oil showed improvements in T-AOC, SOD, GSH-Px, and MDA compared with the control group (*p* < 0.05 or *p* < 0.01 or *p* < 0.001), and there were significant differences between WOC high group and pure walnut oil group (*p* < 0.05) in MDA result. But there were no significant differences found among WOC, VC, and pure walnut oil groups in other results.

**Fig. 6:**
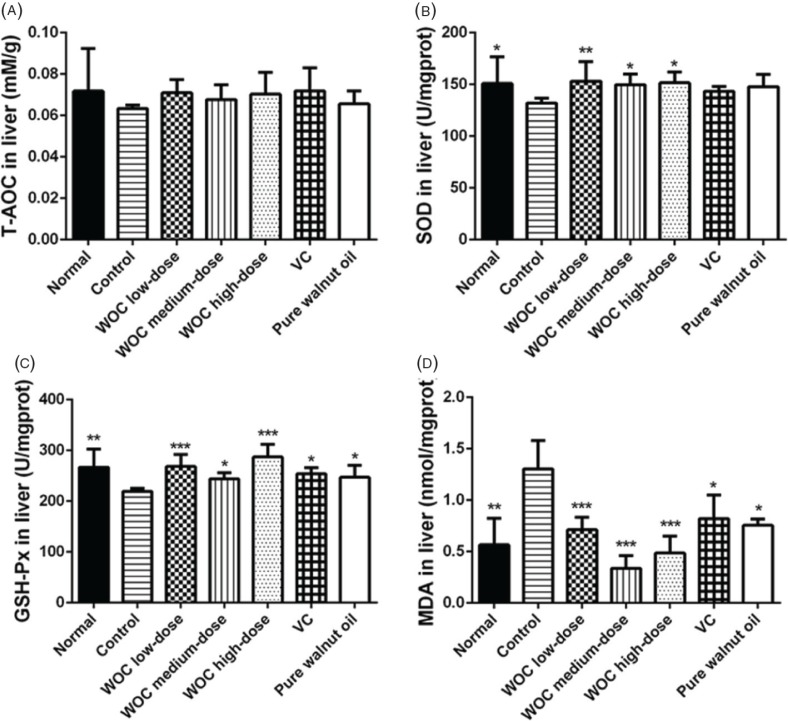
Biochemical indicators in liver. (A) Effects of WOC on T-AOC; (B) Effects of WOC on the activities of SOD; (C) Effects of WOC on the activities of GSH-Px; (D) Effects of WOC on the contents of MDA. *p < 0.05, **p < 0.01,*p < 0.001 versus the control group.

As shown in the result of liver (Fig. 6), there were no significant differences among groups in T-AOC. In WOC groups, the SOD and GSH-Px were significantly higher than the control group and the MDA lower than that in the control group (*p* < 0.05 or *p* < 0.01 or *p* < 0.001). There were also significant differences between the control group and the positive control groups in the results of GSH-Px and MDA (*p* < 0.05) but not in the SOD result. Moreover, the improvement levels of a high dose of WOC was higher than VC and pure walnut oil in GSH-Px (*p* < 0.05), also the MDA level in WOC medium group was lower than positive control groups (*p* < 0.05 or *p* < 0.01).

**Fig. 7:**
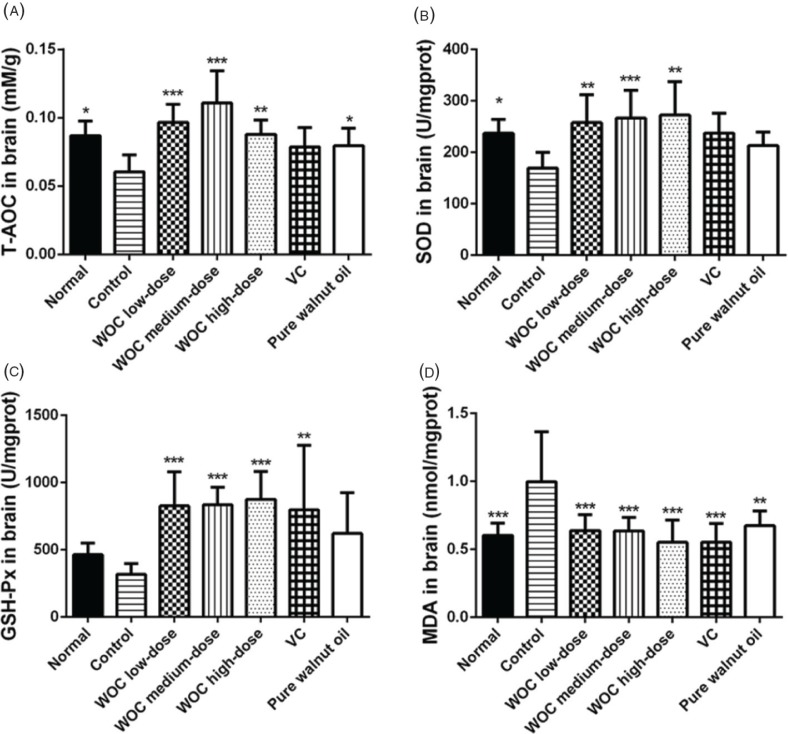
Biochemical indicators in brain. (A) Effects of WOC on T-AOC; (B) Effects of WOC on the activities of SOD; (C) Effects of WOC on the activities of GSH-Px; (D) Effects of WOC on the contents of MDA. *p < 0.05, **p < 0.01,*p < 0.001 versus the control group.

For the result of brain (Fig. 7), the T-AOC levels and the activities of SOD and GSH-Px in WOC groups were significantly higher than that in the control group. The contents of MDA decreased significantly in WOC groups compared with the control group (*p* < 0.01 or *p* < 0.001). Moreover, the T-AOC levels in the WOC medium group were significantly higher than that in the positive control groups (*p* < 0.001).

### Expression of HO-1, iNOS, and Klotho in liver and brain

**Fig. 8:**
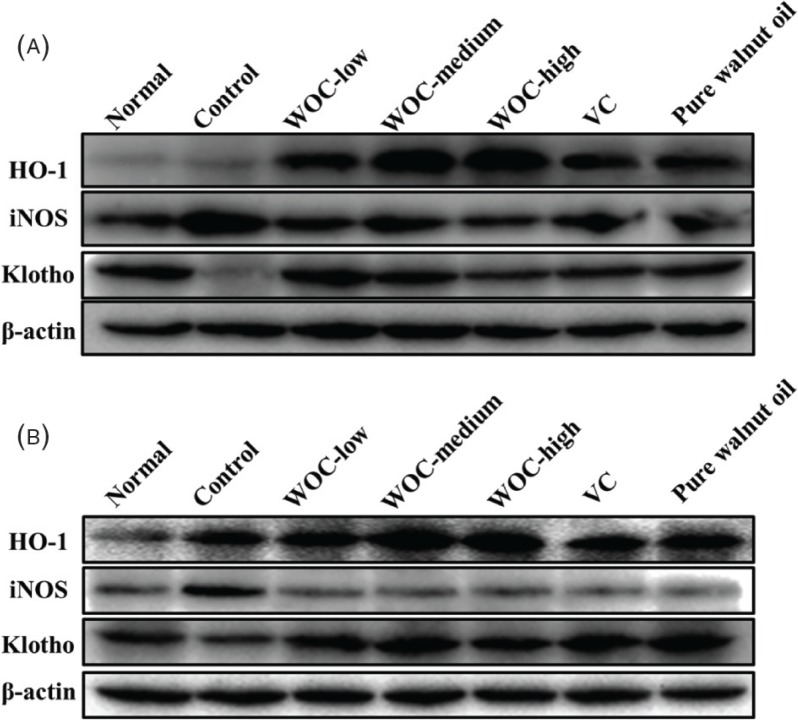
Western blotting assays of HO-1, iNOS, and Klotho. (A) The expression of HO-1, iNOS, and Klotho in liver; (B) The expression of HO-1, iNOS, and Klotho in brain.

In the results of western blotting (Fig. 8), the expression of HO-1 in D-gal-induced aging mice was significantly increased compared with the normal group. Mice treated with WOC, VC, and pure walnut oil all exhibited the higher upregulation of HO-1 than the control group and the enhancements of WOC were higher than VC and pure walnut oil. iNOS found high expression in the control group. WOC, VC, and pure walnut oil could obviously downregulate the expression of iNOS both in liver and brain. Mice in the control group showed extremely low expression of Klotho, but WOC, VC, and pure walnut oil differently upregulated the expression of Klotho in liver and brain.

## Discussion

Aging is an inevitable process which can lead to oxidative stress and affect the function of kidney, brain, liver, and so on (21–24). In recent years, discovery of many antioxidant ingredients has made it possible to delay senescence, and finding effective anti-aging foods or drugs has become a hot topic. Many plants and plant extracts are rich in bioactive constituents and have been widely used in pharmaceutical application and research; thus, they are potential in the screening of antioxidant drugs. In this study, we investigated the compound capsule of walnut oil and grape seed extract to verify its antioxidant capacity and lay basis for its daily use by elderly people.

Generally, in this study, we investigated the antioxidant effects of WOC *in vitro* and *in vivo*. The DPPH scavenging test was selected to explore the antioxidant capacity of WOC *in vitro*. The biochemical indicators such as T-AOC, SOD, GSH-Px and MDA in serum, liver and brain were detected to evaluate the antioxidant of WOC *in vivo*. Also, the western blotting assays were employed to detect some proteins which were closely related to oxidative stress and aging. Moreover, the histopathologic examination was observed to discuss the effects of WOC on tissues.

The DPPH scavenging test showed that the scavenging rate of VC was much higher than WOC, but it was the opposite in *in vivo* research.The reason might be that the instability of VC led to the low bioavailability *in vivo*. Instead, the bioactive ingredients of WOC might have higher bioavailability.

The dose of D-gal covered a wide range wherein the minimal dose could be 50 mg/kg (25), while the higher dose could be 1,250 mg/kg (26). The modeling period usually ranges from 6 to 8 weeks. In this research, the aging model was established by 1,000 mg/kg D-gal for 8 weeks. Different indicators were selected to evaluate the antioxidant effects of WOC *in vivo*. T-AOC could reflect the T-AOC of the body. The activity of SOD and GSH-Px and the content of MDA are closely related to aging process and oxidative stress (27–29). Enzymes of SOD and GSH-Px are important antioxidases with strong ability of eliminating free radicals (27, 30). The MDA can reflect the extent of lipid peroxidation and attacks from free radicals to tissues and cells (31). In the results, WOC could obviously increase the levels of T-AOC and the activities of SOD and GSH-Px. It could also decrease the contents of MDA.

HO-1 is an oxidative, stress-induced enzyme belonging to the HO family, which has strong antioxidant capacity (32, 33). Normally, the expression of HO-1 is low in normal organs, such as brain, and high only in the spleen and liver(34). However, the expression of HO-1 will be upregulated under the condition of oxidative stress (35). In the results, the expression of HO-1 in the aging model group was higher than the normal group. WOC, VC, and pure walnut could obviously upregulate the expression of HO-1 and thus enhance the antioxidant capacity.

iNOS belongs to a family of enzymes called nitric oxide synthase (NOS), which is closely related to the production of nitric oxide (NO) (36). NO plays contrasting roles in living organism. It is an important host defense effector in the immune system. It is also a free radical that plays an important role in pathological processes, especially in inflammatory disorders (37–39). In many systems, the oxidative stress could increase cytokine-mediated iNOS expression (40), which is accompanied by increasing production of NO, and the NO could react with superoxide to produce the strong oxidant peroxynitrite, which in turn can increase lipid peroxidation (41). In our results, WOC could alleviate the oxidative stress by obviously decreasing the expression of iNOS.

Klotho is an anti-aging gene that was first discovered in mice by Kuro-o et al. in 1997. Deficiency of Klotho leads to a syndrome resembling aging, including a short lifespan; stunted growth and kyphosis; vascular calcification and atherosclerosis; osteoporosis; pulmonary emphysema; cognitive impairment; deafness; and atrophy of skin, muscles, gonads, and many other organs (42). Conversely, an overexpression of klotho could extend life span (43). Moreover, studies also indicated that Klotho worked as an important factor in the regulation of oxidative stress, cell proliferation, and apoptosis (44, 45). Our results showed that WOC could upregulate the expression of Klotho and protect against the damages of oxidative stress.

All results showed WOC could enhance the antioxidant capacity of the body, but the improvements did not exhibit a dose-dependent behavior. We inferred that there might be two reasons: (1) The dose gradient we designed was small and so there were no obvious differences among WOC groups; (2) The improvements reached a maximum at a low dose of WOC, but the effects did not enhance with an increase in dose. The expression of proteins HO-1, iNOS, and Klotho were related to many factors and could be regulated through different pathways (41, 46–47). So the mechanism of how WOC regulates the expression of these proteins needs further studies. And, the data of weight changes in mice showed that the growth rate in the WOC group was lower than that in the normal group, leading to the inference that WOC might have a weight-loss function.

## Conclusion

In conclusion, WOC could improve the antioxidant capacity in aging mice induced by D-gal through increasing the activities of antioxidant enzymes, decreasing the contents of MDA, and regulating the expression of oxidative stress–related proteins. Our study suggests that WOC can be used as a promising anti-aging and weight-loss food.
